# A Hybrid Nanocomposite Based on the T-Shaped Carbon Nanotubes and Fullerenes as a Prospect Material for Triple-Value Memory Cells

**DOI:** 10.3390/ma15228175

**Published:** 2022-11-17

**Authors:** Semyon G. Levitsky, Vladislav V. Shunaev, Olga E. Glukhova

**Affiliations:** 1 Department of Physics, Saratov State University, 410012 Saratov, Russia; 2 Institute for Bionic Technologies and Engineering, Sechenov University, 119991 Moscow, Russia

**Keywords:** carbon nanostructure, T-junction, triple-value memory cell, electric field, charge transfer

## Abstract

Relying on empirical and quantum chemical methods, a hybrid nanocomposite based on the T-shaped carbon nanotube (CNT) junction and internal fullerene C_60_ is proposed as a potential triple-value memory cell. The T-shaped CNT provides three potential wells where the internal fullerene can be located. The fullerene can move between these wells under the periodic external electric field, whose strength and frequency parameters are identified. The process of the fullerene’s motion control corresponds to the memory cell write operation. The read operation can be realized by determining the fullerene’s position inside the CNT by estimation of the charge transfer between a fullerene and the CNT’s walls. Calculations took into account such external factors as temperature and air environment.

## 1. Introduction

Low-dimensional structures are the basic element of next-generation devices of various spheres [1,2,3,4]. Nonvolatile memory is one of the most widely used memory devices in modern micro- and nanoelectronic applications. Carbon nanomaterials (CNs) are congenitally nonvolatile. Due to molecular binding forces, CNs can retain their atomic meshes for a long time without external power. For example, Gervasi reported that carbon nanotubes (CNTs) serve data during hundreds of years, even under extreme temperatures in the range from −55 to 300 °C [5]. Field effect transistors (FETs) are often applied as a fundamental element of nonvolatile flash memory. Hossain and Chowdhury proposed a new flow gate transistor where superconductive graphene nanoribbons work as a channel and CNTs with good capacity retention are used as a floating gate [6]. Sun et al. designed three-dimensional memory circuits based on the CNT FET that have increased integration density by up to 82.92% in comparison to traditional 2D memory arrays [7]. It was shown that modification of the CNT can improve the charge storage properties in memory cells. For example, oligomer hydrate crystallization enlarged the memory window of CNT-FET by four orders of the current’s magnitude [8].

Three-value logic was first introduced by Lukasiewicz as the simplest example of the many-value logic [9]. Systems based on the three-value logic can improve performance of current processors and increase bit handling capability as well as reduce the number of active memory blocks in a device [10]. Mousavi et al. implemented the triple-value memory cell on the base of the CNT FET [11]. Voltage differs in this cell from 0 to 0.9 V, and zero voltage stands for the logic “0”, 0.45 V for the logic “1” and 0.9 V for the logic “2”. Hellkamp and Nepal designed a three-valued ternary content addressable memory cell on the base of ambipolar carbon nanotube transistors, with improved savings of up to 31% in comparison to traditional designs [12]. Khasanvis et al. proposed a multistate volatile memory circuit on the base of a graphene nanoribbon crossbar and showed that the phase difference between the standing waves of individual nanoribbons could be controlled by changing the potential between top and bottom layers of nanoribbons [13]. Such devices can be also applied as multiscale memory elements.

Hybrid nanocomposites based on carbon materials have proven themselves as performant multifunctional materials [14,15,16,17,18]. For synthesis of such nanocomposites, both ex situ and in situ methods are used [19]. Among them, the sol-gel method [20,21,22], hydrothermal treatment [23,24], chemical vapor deposition (CVD) on catalyst nanoparticles [25,26], and electrostatic self-assembly [27,28] are in great demand nowadays. Carbon heterojunctions have been recently considered as prospect elements for photoelectrochemical/optoelectronic devices, solar cells, and batteries [29,30]. Among them, the focus is on carbon nanopeapods representing CNTs with internal fullerenes or other molecules. Earlier, Okazaky comprehensively reviewed existing methods for the preparation of CNTs with encapsulated fullerenes C_60_, C_70_ and C_82_ and their derivatives [31]. It was shown that encapsulation of a fullerene into a CNT’s cavity modifies its electronic properties [31,32]. The patterns of dynamic behavior of the C_60_ fullerene inside the CNT were studied by methods of molecular dynamics. Lee at al. studied the C_60_@CNT composite at 4000, 5000 and 6000 K and confirmed that thermal conductivity in the composite is mainly transmitted through a C_60_ fullerene [33]. Jeong and Kim systematically explored the influence of different loads on the behavior of the CNT filled with C_60_ fullerenes and found that the filling of the CNT with C_60_ fullerenes strengthened the structure under uniaxial loading [34]. Kuo et al. confirmed that carbon nanopeapods have a higher adhesive strength than CNTs [35]. Finally, it was shown that the motion of the K^+^@C_60_ complex in the CNT can be controlled by the external electromagnetic field at frequencies from 132 GHz to 3 THz [36]. Herewith, the position of the internal complex significantly changes the I-V curve of the nanopeapod due to the change of the electron transfer.

The synthesis and exploration of CNT T-junctions have taken place over the last few decades [37,38], and modern techniques promise the successful fabrication of well-defined T-shaped CNTs in the laboratory [39]. The aim of this work is to design a three-value memory cell on the base of the T-shaped CNT with encapsulated fullerene C_60_. The topology of the CNT with T-junctions assumes the presence of three potential wells that are essential for the three-valued logical element. The position of the internal fullerene in one of these wells stands for three different logic states. The movement of a fullerene between potential wells is held by the external electric field.

## 2. Materials and Methods

The dynamics of the fullerene inside the CNT were determined by classical molecular dynamics (MD) that numerically integrated Newton’s equations of motion by the Verlet algorithm using a time step of 0.1 fs. All MD calculations were carried out at the temperature of 300 K, which was controlled by a Berendsen thermostat [40]. Since the considered structures consisted of ≈3100 atoms, the semi-empirical method AIREBO [41] developed for hydrocarbons was chosen for determination of forces applied to atoms. Taking into account the external electric field, the additional radiation force and radiation losses were introduced at the stage of force calculations [42]. Long-range van der Waals interactions between atoms whose interatomic distance exceeded the maximal C-C bond length were defined by the Lennard-Jones potential [43]. The ground states of the considered models were found by minimization of their potential energy.

The quantum calculations were performed by the self-consistent-charge density-functional tight-binding method (SCC DFTB) [44]. The basic set trans3d-0-1 was applied to describe the interaction between C, Fe and Co atoms [45], and the set 3ob-3-1 described the interaction between C and K atoms [46]. Optimization was performed at the electronic temperature of 300 K, with 2 × 2 × 2 Monkhorst-Pack Brillion zone sampling. SCC tolerance was equal to 1 × 10^−5^. The distribution of charge at the atoms was calculated by the Mulliken procedure [47].

The seamless T-junctions of CNT were built by the original method for generating atomistic models of multi-branched and arbitrarily shaped seamless junctions of carbon nanostructures [48].

## 3. Results

### 3.1. Controlled Motion of the Internal Fullerene between Three Wells Inside the CNT (Write Operation)

**Figure 1:**
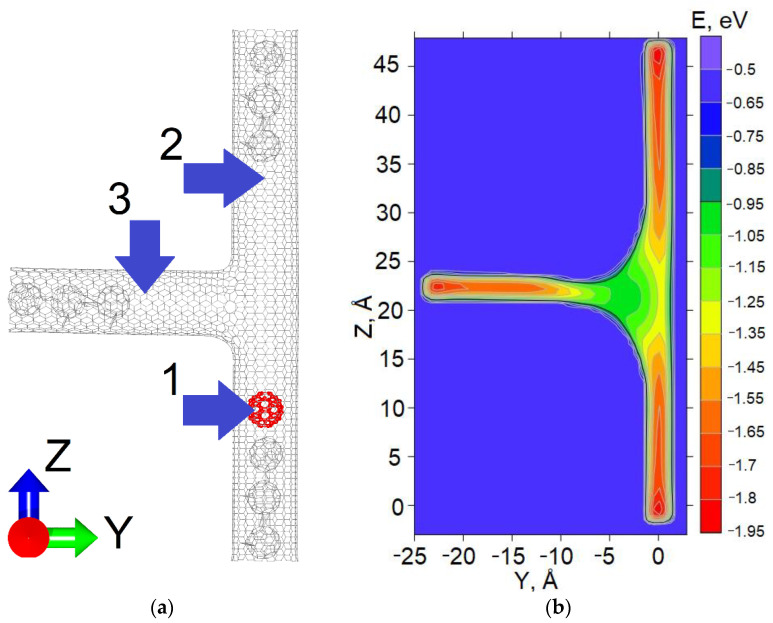
The hybrid carbon nanostructure based on the T-shaped CNT and fullerene C_60_: (**a**) atomic structure: the fullerene C_60_ marked in red is free and can move inside the CNT between potential wells «1», «2» and «3», and chains of three fullerenes are chemically bonded to the CNT walls; (**b**) the energy profile of the van der Waals interaction of the free fullerene and the CNT walls.

The considered physical model represented the seamless T-junction of CNT (10,10) with three chains of fullerene C_60_ chemically bonded to CNT walls near its edges (Figure 1a). These fullerene chains created potential wells and forbade elimination of the free fullerene C_60_ (marked in red) from the cavity of CNT. The position of the free fullerene in one of the potential wells corresponded to three possible logic states of the considered memory cell. Since the main interaction between the free fullerene and the CNT walls was van der Waals, the energy profile was calculated based on the Lennard-Jones potential (Figure 1b). The depth of the potential well “1” was equal to −1.929 eV, “2” to −1.913 eV and “3” to −1.911 eV. The difference between energies can be explained by randomness of optimization. According to the set task, it is possible to change these values by varying the arrangement of fullerene chains.

It was decided to move the fullerene between the potential wells by the external periodic electric field. At the first stage, the natural frequency of the free fullerene was found after the MD computation of the considered hybrid carbon nanostructure under the temperature of 300 K. The application of the Fast Fourier Transform to the trajectory of the free fullerene’s center mass revealed the spectral characteristic of the frequency that showed the intensity peak at 0.457 THz, as in ref. [36]. So, the application of the electric field with this frequency is justified.

At the next stage before the simulations, the electron charge −1 was uniformly distributed between the CNT walls and the electron charge +1 between the atoms of the free fullerene, so the system remained neutral. This was done to consider the influence of the radiation force induced by the electric field. The distribution of +1e charge at the atoms of the free fullerene corresponded physically to encapsulation of a metal’s cation (K^+^, Fe^+^, Co^+^, Ni^+^). It should be noted that such cations as Fe^+^, Co^+^ and Ni^+^ are usually nonexistent, so their encapsulation into the fullerene cage is a difficult task. To enlarge the amount of charge, such cations as Mg^2+^ and Al^3+^ can be used. In the result of numerous simulations, the strength and the direction of the electric field required for the motion of the free fullerene between potential wells were determined (Table 1). It can be seen that the strength of the electric field was equal to 1.6 or 1.65 V/nm, dependent on the initial potential well and the applied direction. The average time of the fullerene’s motion was 5 ps. Note that the increase in the electric strength will also lead the fullerene to reach the required well, but there is no guarantee that it will be kept there.

It should be also noted that simulations were carried out under the temperatures in the range from 184 to 330 K, which correspond to the highest and the lowest temperatures registered on Earth. The temperature’s value almost doesn’t affect the time of the fullerene’s motion between the potential wells. The corresponding video files of the movements from the well “1” to the well “2” are specified in the Supplementary Materials. Under the temperature of 184 K, the fullerene complex reached well “2” after 9 ps; then, in the range of 9 ÷ 53 ps, it oscillated near the potential well and then remained there (see Video S1). Under the temperature of 257 K, the fullerene complex reached well “2” after 9 ps; in the range of 9/74 ps, it oscillated near the potential well and then remained there (see Video S2). Under the temperature of 330 K, the fullerene complex reached well “2” after 9 ps; in the range of 9/59 ps, it oscillated near the potential well and then remained there (see Video S3). So, the entire range of possible Earth temperatures gave satisfying results.

### 3.2. Detection of the Current Fullerene’s Position (Read Operation)

The SCC DFTB method with application of charge self-consistency is much more energy consuming than the AIREBO and is not often used for MD simulations of atom structures. However, it allows calculations for many more types of atoms than the AIREBO as well as calculations of some important electronic parameters such as the charge transfer. Naturally, calculations by the SCC DFTB are more precise and reliable. For example, there is no need to distribute the charge 1e between fullerene atoms since we can encapsulate a metal’s ion into the fullerene’s cavity. The encapsulation of potassium, aluminum, magnesium, ferrum and cobalt atoms into C_60_ can be performed experimentally [49,50,51]. To reach the maximal effect of the external electric field, the charge transfer between the fullerene mesh and an encapsulated ion should be the biggest. Our calculation showed that after encapsulation, the Fe atom reports the fullerene the charge of −0.17e, Mg atom is −0.05e, Al atom is −0.22e, Co atom is −0.37e and K atom is −0.96e. Thus, the K@C_60_ will have the biggest response to the external field; all subsequent calculations are provided for this endohedral complex.

**Figure 2:**
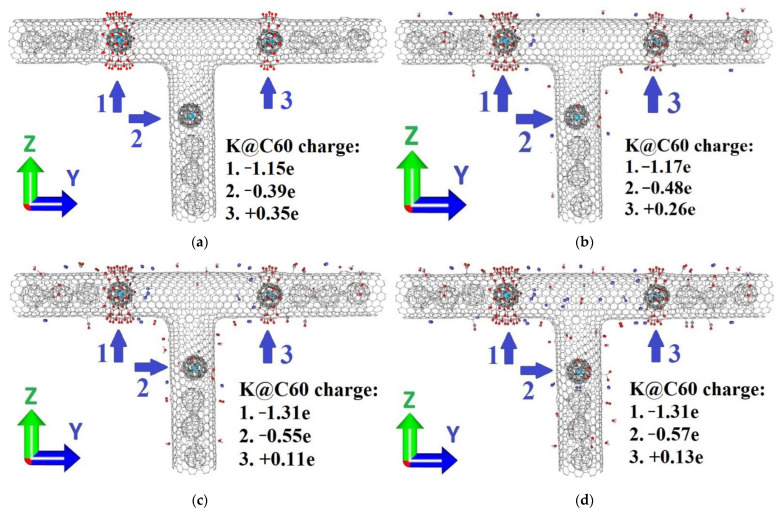
The atomic structure of the hybrid carbon nanostructure based on the CNT and the endohedral complex K@C_60_ modified with epoxy groups: the left edge is decorated with six rings of oxygen (well “1”), the right edge with 3 rings (well “2”), the bottom edge was pure (well “3”): (**a**) concentration of air molecules 0%; (**b**) 0.019%; (**c**) 0.038%; (**d**) 0.057%.

As was mentioned, all three potential wells inside the considered hybrid CNT had almost equal energy depth (Figure 1a), and the location of the fullerene in one of them had little variation in the properties of the hybrid nanostructure, which complicated the read operation. To overcome this problem, we decided to modify two of the edges with oxygen epoxy groups. Note that the left edge was oxidized with six rings of epoxy groups, while the right edge was oxidized with three (Figure 2a). Selective oxidation of the CNT has been under development for a long period, so this process is easily realized [52,53,54]. The fullerene’s position affected the charge transfer in the structure significantly. In well “3” (with a non-oxidized edge) the K@C_60_ complex reported 0.35e to the tube’s wall. With the presence of the oxygen at the tube’s walls, the complex acted as an acceptor: it received 0.36e in well “2” and 1.15 e in well “3”.

Since the suggested device is assumed to work in an air environment, we considered three different cases of ratio between atoms of air molecules and the composite: 0.019% (Figure 2b), 0.038% (Figure 2c) and 0.057% (Figure 2d). For this goal, such air components as CO_2_, N_2_, CO and H_2_O were attached to the surface of the considered hybrid nanocomposite. Note that CO and CO_2_ molecules formed a covalent bond with the carbon mesh while N_2_ and H_2_O molecules interacted with the composite by van der Waals forces. N_2_, CO and H_2_O molecules acted as a donor of charge, and the CO_2_ group as an acceptor. The presence of air molecules slightly changed the amount of the transferred charge (Table 2). With the growth of air concentration up to 0.038%, the excess of charge in the K@C_60_ complex increased from −1.15e to −1.31e for position “1” and from −0.39e to −0.55e for position “2”. For position “3”, the lack of charge in the K@C_60_ complex was decreased from 0.35e to 0.11e. The following growth of air molecule concentration up to 0.057% resulted in little change to the charge transfer, so we assume the amount of charge transfer won’t change with the following increase of air molecule concentration.

Thus, the read operation can be provided by evaluating the value of the charge on the tubes’ walls. Another variant is measurement of I–V curves as proposed in ref. [36]; however, this is a difficult task for modeling because of a large number of atoms.

## 4. Conclusions

The hybrid nanostructure on the base of the T-shaped CNT and fullerene C_60_ were proposed as a three-valued memory cell. Under normal conditions, the free fullerene can take place in one of the three potential wells that corresponds to logic states “1”, “2” and “3”. By means of the AIREBO method, the strength, frequency and directions of the electric field required for controlling the motion of the fullerene between these logic states were found. The frequency equaled 0.457 THz, and the strength of the electric field was 1.6 or 1.65 V/nm, depending on the initial fullerene position. The process of the fullerene’s movement between the wells corresponds to the memory cell write operation. The memory cell read operation corresponds to the estimation of the charge transfer at the tube’s walls, depending on the fullerene’s position. For this goal, CNT’s atoms around the first potential well were decorated with six rings of epoxy groups and the second one by three rings, and the atoms around the third well are not oxidized. The Mulliken charge on the free fullerene in state “1” equaled −1.12 e, in state “2” equaled −0.39 e, and in state “3” equaled 0.35e. The presence of air molecules (CO_2_, N_2_, CO and H_2_O) shifts the amount of charge transfer to −1.31e in state “1”, to −0.55e in state “2” and to 0.31e in state “3” when the ratio of air atoms to composite atoms equals 0.057%. The following increase of air molecule concentration has little effect on the amount of charge transfer. The series of molecular dynamics experiments proves that due to the unique thermal stability of carbon nanomaterials, the proposed logic circuit can operate at any possible Earth temperature (from 184 to 330 K). We assume that topological defects will not significantly affect the work of the suggested memory cell. Note that diameter of fullerene C_60_ is ~0.7 nm and the diameter of CNT (10,10) is ~1.3 nm. Of course, another diameter of CNT as well as another type of a fullerene will affect the amount of charge transfer and values of energy in potential wells, which will change the parameters of electric field required for successful movement of fullerenes between logic states. However, we suppose for the diameter ratio 0.7:1.3, the results will be the same. So, the proposed three-valued memory cell on the base of the T-shaped CNT and fullerene C_60_ can be important elements for nano-RAM—the logical replacement of DRAM.

## Figures and Tables

**Table 1:** The strength (V/nm) and the direction of (X;Y;Z) of the electric field required for the free fullerene’s motion between potential wells “1”, “2” and “3”.

Number of Well	1	2	3
**1**	-	1.6 (0; 0; 1)	1.65 (0; −0.75; 1)
**2**	1.6 (0; 0; −1)	-	1.65 (0; −0.75; −1)
**3**	1.65 (0; 1; −0.75)	1.6 (0; 1; 0.75)	-

**Table 2:** The amount of charge transferred to K@C_60_ complex dependent on concentration of air molecules near the composite.

Well/Concentration	0%	0.019%	0.038%	0.057%
I	−1.15	−1.17	−1.31	−1.31
II	−0.39	−0.48	−0.55	−0.57
III	+0.35	+0.26	+0.11	+0.13

## Supplementary Materials

The following supporting information can be downloaded at: https://www.mdpi.com/article/10.3390/ma15228175/s1, Video S1: The movement of the free fullerene from well 1 to well 2 under 184 K; Video S2: The movement of the free fullerene from well 1 to well 2 under 257 K. Video S3: The movement of the free fullerene from well 1 to well 2 under 330 K.
